# GCY-35/GCY-36—TAX-2/TAX-4 Signalling in O_2_ Sensory Neurons Mediates Acute Functional Ethanol Tolerance in *Caenorhabditis elegans*

**DOI:** 10.1038/s41598-018-20477-z

**Published:** 2018-02-14

**Authors:** Yuan-Hua Chen, Chang-Li Ge, Hong Wang, Ming-Hai Ge, Qing-Qin He, Yu Zhang, Wei Tian, Zheng-Xing Wu

**Affiliations:** Key Laboratory of Molecular Biophysics, Ministry of Education, and Department of Biophysics and Molecular Physiology, College of Life Science and Technology, Huazhong University of Science and Technology, Wuhan, 430074 P.R. China

## Abstract

Ethanol is a widely used beverage and abused drug. Alcoholism causes severe damage to human health and creates serious social problems. Understanding the mechanisms underlying ethanol actions is important for the development of effective therapies. Alcohol has a wide spectrum of effects on physiological activities and behaviours, from sensitization to sedation and even intoxication with increasing concentrations. Animals develop tolerance to ethanol. However, the underlying mechanisms are not well understood. In *Caenorhabditis elegans*, NPR-1 negatively regulates the development of acute tolerance to ethanol. Here, using *in vivo* Ca^2+^ imaging, behavioural tests and chemogenetic manipulation, we show that the soluble guanylate cyclase complex GCY-35/GCY-36—TAX-2/TAX-4 signalling pathway in O_2_ sensory neurons positively regulates acute functional tolerance in *npr-1* worms.

## Introduction

Ethanol is a widely used beverage. Its abuse causes severe damage to human health and creates serious social problems, including an increased risk of cardiomyopathy, liver complaints, traffic accidents, loss of work and learning problems^1–3^. Alcohol alters the behaviour of both invertebrates and vertebrates. Upon exposure to ethanol, humans, rodents and *Drosophila* exhibit responses to ethanol ranging from disinhibition and euphoria at low doses to incoordination, lethargy and even death at higher doses^4,5^. In *Caenorhabditis elegans* (*C. elegans*), ethanol intoxication suppresses locomotion, swimming and egg laying^6^. A number of identified genes contribute to alcoholism^7,8^. Ethanol acts on a wide spectrum of target sites. Ethanol inhibits NMDA^9^, AMPA^10^ and kainate^11^ receptors. It enhances GABA_A_^12^ and glycine receptors^13,14^, potentiates ion currents through the ligand-gated ion channel of the 5-hydroxytryptamine-3 (5-HT_3_)-type serotonin receptor^15,16^ and modulates the neuronal acetylcholine receptor^17^. Ethanol acts on a calcium-activated large conductance BK potassium channel, SLO-1, to inhibit neuronal activity and plays a key role in alcohol intoxication in the nematode *C. elegans*^6,18^.

Animals develop resistance to toxins. There are two general routes for evolving resistance to a toxin. One is to evolve a reduced sensitivity to the toxin’s effects. The second route is to minimize the amount of toxin that reaches the target organ or tissue. The underlying mechanisms include detoxification, excretion, sequestration and reduced absorption^19^. *C. elegans* develops tolerance to ethanol. Multiple genes that control acute sensitivity to ethanol have been identified in *C. elegans*^7,20^. The cAMP-PKA signal transduction pathway functions in ethanol tolerance in *Drosophila*^5^. nNOS/NO/cGMP/cGKII signalling positively regulates sensitivity to the sedative hypnotic effects of a high ethanol dose in mice^21,22^. However, it is still important to identify novel genes and signalling pathways that are involved in acute ethanol tolerance.

Due to feasible genetic and neuronal manipulations, rich genetic tools and vertebrate neuronal protein homologues, the nematode *C. elegans* is a useful model animal to identify novel ethanol target sites and molecular bases for ethanol tolerance. Ethanol affects *C. elegans* physiological activities and behaviours. The worms exhibit uncoordinated behaviour and intoxication when exposed to 100 mM (~0.6%) and 500 mM (~3%) ethanol, respectively^6^. Several molecules, including SLO-1, NPR-1 and the switching defective/sucrose nonfermenting (SWI/SNF) chromatin-remodelling complex, are involved in modifying acute tolerance to ethanol in *C. elegans*^6,23,24^. NPR-1 negatively modulates acute ethanol tolerance. The *C. elegans* strains with a lower function or loss-of-function alleles recover more quickly from exposure to ethanol than N2 animals^23^. N2 animals express a high-activity form of the NPR-1 receptor that suppresses output of a circuit detecting hyperoxia^25^ mainly by blocking output of RMG interneurons^26^. Here, using *in vivo* Ca^2+^ imaging, behavioural tests and chemogenetic experiments, we present evidence that suggests that the GCY-35/GCY-36—TAX-2/TAX4 signalling pathway in O_2_ sensory neurons URX, AQR and PQR, mediates the ethanol excitatory effect on these neurons and functions in acute functional ethanol tolerance in *npr-1* animals.

## Results

### GCY-35/GCY-36—TAX-2/TAX-4 signalling pathway is involved in acute functional ethanol tolerance in *npr-1* worms

**Figure 1:**
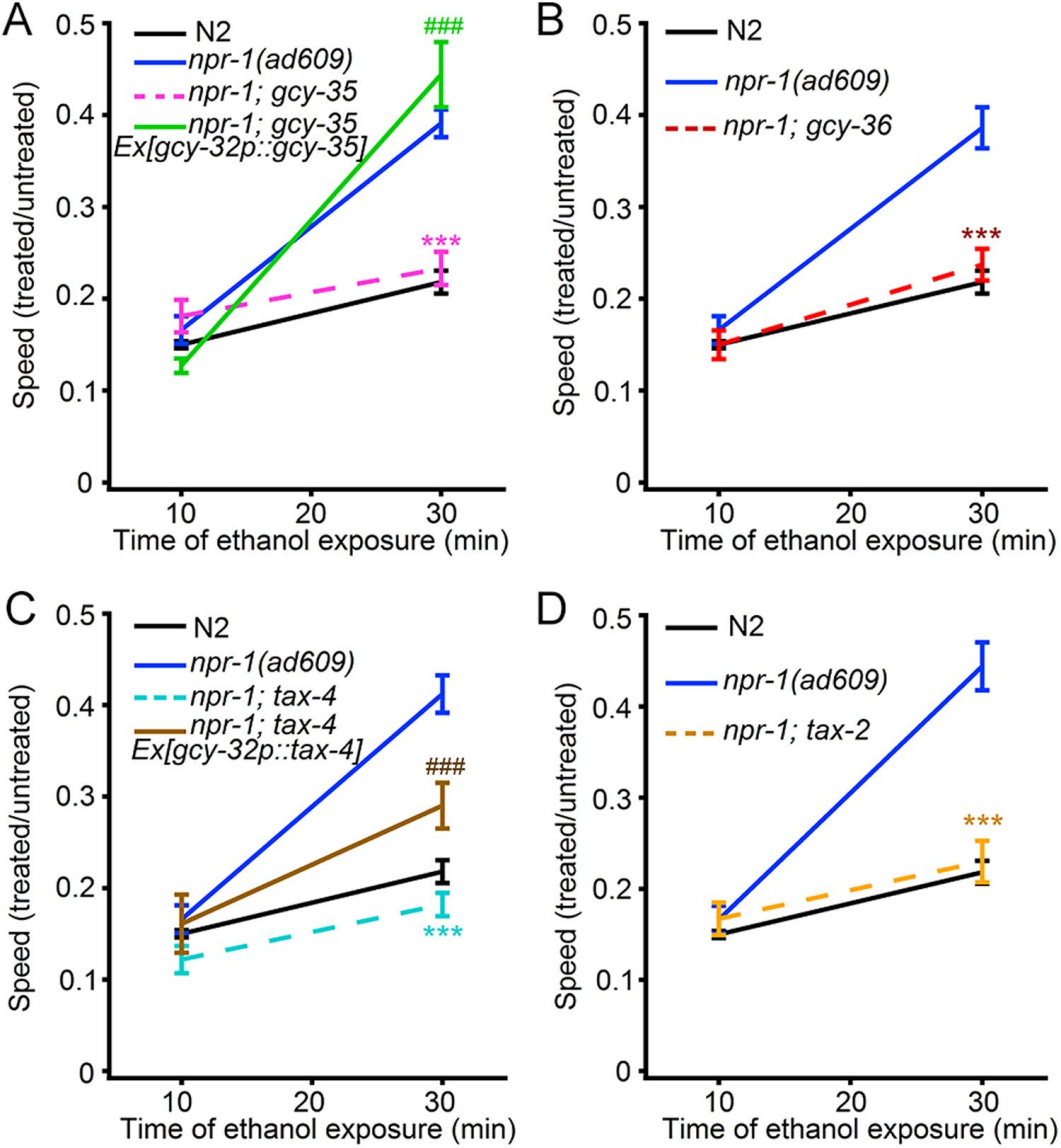
The genes encoding GCY-35/GCY-36 and TAX-2/TAX-4 are required for  acute functional ethanol tolerance in *npr-1* worms. (**A**–**D**) The relative locomotion speed (ethanol treated/untreated) at 10 and 30 min after ethanol exposure in (**A**) wild type N2*, npr-1*(*ad609*), *npr-1*(*ad609*); *gcy-35*(*ok769*) mutant and the *gcy-32p*::*gcy-35* transgene, the same worm strains are not listed hereinafter, (**B**) *npr-1*(*ad609*); *gcy-36*(*db42*), etc., (**C**) *npr-1*(*ad609*); *tax-4*(*p678*), *gcy-32p*::*tax-4* transgene, etc., (**D**) *npr-1*(*ad609*); *tax-4*(*p678*), etc. The data are analyzed by two-way ANOVA. In (**A**), *F*_Time_ (1, 75) = 75.63, *P* < 0.0001; *F*_genotype_ (3, 75) = 7.667, *P* < 0.0001; *F*_genotype * Time_ (3, 75) = 11.86, *P* = 0.0002. In (**B**), *F*_Time_ (1, 49) = 50.35, *P* < 0.0001; *F*_genotype_ (2, 49) = 12.53, *P* < 0.0001; *F*_genotype * Time_ (2, 49) = 8.229, *P* = 0.0008. In (**C**), *F*_Time_ (1, 58) = 61.91, *P* < 0.0001; *F*_genotype_ (3, 58) = 16.08, *P* < 0.0001; *F*_genotype * Time_ (3, 58) = 8.584, *P* < 0.0001. In (**D**), *F*_Time_ (1, 43) = 55.93, *P* < 0.0001; *F*_genotype_ (2, 43) = 18.66, *P* < 0.0001; *F*_genotype * Time_ (2, 43) = 16.26, *P* < 0.0001. In (**A**–**D**), significant posttest comparisons with *npr-1(ad609)* at any given time point are indicated with ***p ≤ 0.001. In (**A**,**C**), significant posttest comparisons with double mutant strain at any given time point are indicated with ^###^*p* ≤ 0.001.

N2 and *npr-1* animals show different rates of recovery from ethanol exposure^23^. A previous work has shown that in N2 animals, the high-activity form of the NPR-1 receptor blocks output from the AQR, PQR, and URX O_2_ sensing neurons^25^. We speculated that the different rates at which N2 and *npr-1* animals recovered from ethanol exposure reflect differences in the functions of this O_2_ sensing circuit. To test this possibility, we examined the behaviour of mutants defective in the function of this circuit, including *gcy-35* and *gcy-36*. We recorded the locomotion of acute ethanol-treated worms on agar plates as described^6,23,27^. In brief, we transferred worms deprived of food for 30 min to the test NGM plates that were bacteria unseeded and ethanol added (final concentration, 500 mM) and then recorded the worms’ movement over 2 min beginning at 10 and 30 min using a CCD camera at 1 frame/s. The relative locomotion speeds (treated/untreated) at 10 min and 30 min of exposure to an exogenous dose of 500 mM ethanol were used as indicators of sensitivity to and acute adaptation or tolerance to ethanol as previous reports suggested^6,23,27^. Our results showed that almost all single gene mutants of *gyc-35*, *gcy-36*, *tax-2* and *tax-4* exhibited ethanol sensitivity and acute functional ethanol tolerance similar to N2 worms (Supplementary Fig. 1). *npr-1*(*ad609*) showed increased acute functional tolerance (Fig. 1A, Supplementary Movies 1–4) as reported previously^23^. Interestingly, *npr-1*(*ad609*); *gcy-35*(*ok769*) double mutants moved slower than *npr-1*(*ad609*), similar to N2 worms at 30 min. Sensitivities to ethanol (indicated by the relative locomotion speed at 10 min) in all strains were similar (Fig. 1A, Supplementary Movies 5 and 6). The gene *gcy-35* is expressed in URX, AQR and PQR sensory neurons^28^. We used the *gcy-32* promoter^29^ to drive *gcy-35* cDNA expression specifically in these three types of neurons in the double mutant. The extrachromosomal expression of *gcy-35* cDNA in these neurons fully rescued the locomotion phenotype in the double mutants (Fig. 1A, Supplementary Movies 7 and 8). The gene *gcy-36* co-expresses with *gcy-35* in URXs, AQR and PQR^28^. GCY-35 and GCY-36 form a soluble guanylyl cyclase (sGC) heterodimer in *C. elegans*^30^. Similar to *npr-1*(*ad609*); *gcy-35*(*ok769*) double mutant, *npr-1*(*ad609*); *gcy-36*(*db42*) double mutant showed a reduced relative speed compared with that in *npr-1*(*ad609*) and similar to that in N2 worms (Fig. 1B). These results suggest that *gcy-35*/*gcy-36* is involved in acute ethanol tolerance in *C. elegans*. The sGC GCY-35/GCY-36 complex catalyses the biosynthesis of cGMP, and the latter acts on the downstream effector, the TAX-2/TAX-4 channel, to play a role in chemosensations in *C. elegans*^25,31–33^. We then examined the role of *tax-2*/*tax-4* in acute functional ethanol tolerance. Our data showed that *npr-1*(*ad609*); *tax-4*(*p678*) and *npr-1*(*ad609*); *tax-2*(*p671*) double mutants manifested a slower speed compared with that of the *npr-1* mutant, similar to that in N2 worms (Fig. 1C,D). The extrachromosomal expression of *tax-4* cDNA driven by the *gcy-32* promoter markedly restored the *npr-1* locomotion phenotype in the *npr-1*(*ad609*); *tax-4(p678)* double mutant (Fig. 1C). Collectively, these results suggest that the GCY-35/GCY-36—TAX-2/TAX-4 signalling pathway likely function in URX, AQR and PQR neurons and is required for the acute tolerance in *npr-1* animals.

NPR-1 and the oxygen-sensitive sGC GCY-35/GCY-36 complex modulate worm locomotion speed in different oxygen levels. *Hawaiian* wild strain CB4856 and *npr-1*(*ad60*9) mutants move faster at high ambient oxygen levels (21%) than the N2 worm does^25,34^. To test the possible impact of high ambient oxygen levels on the locomotion of ethanol-treated worms, we developed a simple device to make a low ambient oxygen level and examined worm locomotion under this condition (Supplementary Fig. 2A, see methods for details). The O_2_ levels were assayed by measuring O_2_-induced quenching of the fluorescent dye Ru(phen)_3_Cl_2_^35^. After infusing a mixed gas of 7% [O_2_] for nearly 12 min, the O_2_-level reduced to 7% in the device (Supplementary Fig. 2B). The *npr-1*(*ad609*) mutant moved slower under low ambient O_2_ levels than in air (Supplementary Fig. 2C) as expected, in agreement with a previous report^34^. This result verifies that this simple device worked well for the tests under a low oxygen level. We recorded the worm locomotion speed under low and high ambient O_2_ levels. Our results showed that the O_2_ levels had no significant impact on relative (Supplementary Fig. 2D) and absolute speeds in all ethanol (500 mM)-treated (Supplementary Fig. 2F) and untreated (Supplementary Fig. 2E) worms at 30 min, although ethanol treatment slowed the worms. These results suggest that the O_2_ levels have no effect on the movement in short-term food-deprived worms, regardless of whether the worms were ethanol treated or untreated.

### Ethanol activates URX and AQR sensory neurons

**Figure 2:**
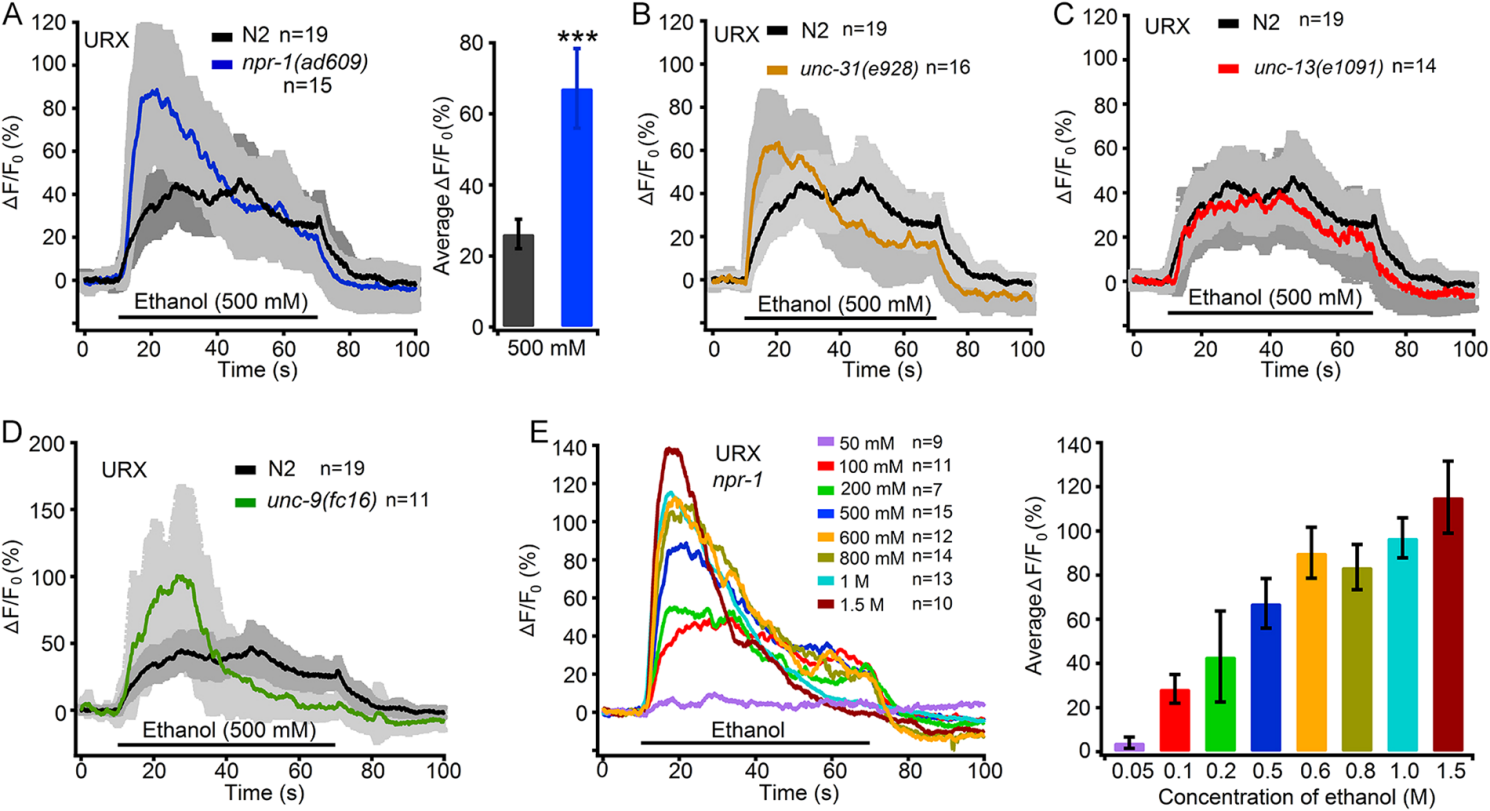
O_2_ Sensory neuron URXs are activated by ethanol. (**A**) Ethanol (500 mM) evoked somal calcium signals of URXs in wild type N2 worm and *npr-1(ad609)* mutant. The left panels show mean intensities of Ca^2+^ signals (in solid traces) with S.E.M. (in grey), the right panels display summaries of the peak (12 s–22 s) signals, and the number of independent tests for each genotype or each concentration of ethanol is indicated, similarly hereinafter. ***p ≤ 0.01 compared with wild type N2 worm. (**B**–**D**) Somal Ca^2+^ levels of URXs in responses to 500 mM ethanol in animals (**B**) *unc-31*(*e928*), (**C**) *unc-13*(*e1091*) and (**D**) *unc-9*(*fc16*) mutants. (**E**) Somal Ca^2+^ levels of URX in responses to ethanol of different concentrations in *npr-1*(*ad609*) mutant.

Our data indicate that the GCY-35/GCY-36—TAX-2/TAX-4 signalling pathway in URXs, AQR and PQR plays a role in acute functional ethanol tolerance in *C. elegans*. A previous study reported that optogenetically activating these three types of neurons induces worm to move faster^34^. We hypothesized that these neurons sense ethanol and mediate the acute functional ethanol tolerance effect. To test this possibility, we used the genetically encoded calcium sensor R-GECO1^36^ combined with a microfluidic device to record *in vivo* somatic calcium responses to ethanol in these neurons. In brief, a transparent polydimethylsiloxane (PDMS) microfluidic device was used to immobilize and expose worms to ethanol stimulation^31,37,38^ (see methods for details). We recorded Ca^2+^ transients of neuronal soma in response to an ethanol concentration of 500 mM, also used in the behavioural assay. As expected, URXs in wild-type N2 worms and *npr-1*(*ad609*) mutants displayed robust Ca^2+^ responses to 500 mM ethanol stimulation, and the responses in *npr-1*(*ad609*) mutants were stronger than those in the N2 worms (Fig. 2A). This may explain why *npr-1* animals show acute functional ethanol tolerance compared with N2 worms. The AQR neuron also displayed robust Ca^2+^ responses to the 500 mM ethanol stimulation (Supplementary Fig. 3). PQR neuron localizes in the worm’s tiny tail. It is not easy to immobilize the worm tail. We did not record the Ca^2+^ response in this neuron. As URXs exhibited more robust Ca^2+^ signals and had almost no delay, we recorded URXs only thereafter.

It is possible that signals transmitted from other neurons result in Ca^2+^ transients in the tested neurons. We thus employed an *unc-13* and *unc-31* lof mutation to validate whether the Ca^2+^ transients in URX are induced by presynaptic neurons. UNC-13 is essential for synaptic vesicle exocytosis and neurotransmitter release^39^, and UNC-31 plays a major role in Ca^2+^-dependent regulative exocytosis of dense-core vesicles and release of neuropeptides^40,41^. The lof mutants were used to analyse the response in sensory neurons under neurotransmission isolation^38,42,43^. In *unc-31*(*e928*) and *unc-13*(*e1091*) mutants, URXs also displayed robust ethanol-elicited Ca^2+^ signals similar to those in N2 worms (Fig. 2B,C). These data indicate that the Ca^2+^ response to ethanol in URXs is not likely a result of chemical neurotransmission from its presynaptic neurons. However, URX neurons connect with RMG and IL2 neurons by gap junctions^44^, and it possible that these electrical synaptic connections cause Ca^2+^ transients in URXs. The innexins (invertebrate connexin analogue) that compose gap-junction channels in URX are not yet known. In virtue of the fact that RMGs and IL2s express *unc-7* and *unc-9*, respectively^45^, and that UNC-7 and UNC-9 form heterotypic channels to mediate electrical synapses^46^, we thus employed *unc-9*(*fc16*) mutants to examine ethanol-elicited Ca^2+^ signals in URXs. Our results showed that URXs in *unc-9(fc16)* displayed even stronger Ca^2+^ transients in response to 500 mM ethanol (Fig. 2D). In summary, these data support that URXs respond to ethanol in a cell-autonomous manner.

Next, we assayed URX Ca^2+^ responses to different concentrations of ethanol in the *npr-1*(*ad609*) mutant. Our data indicated that the Ca^2+^ responses in URXs were well related with the ethanol concentrations; however, they were not well fitted by the Hill function (Fig. 2E). Does ethanol alter oxygen levels in the M13 buffer and then affect URX neurons? We then assayed dissolved oxygen levels in the ethanol-M13 solutions with various ethanol concentrations. Our results showed that ethanol levels did not significantly influence dissolved oxygen levels in the solutions (Supplementary Fig. 4). The above results suggest that ethanol directly activates *gcy-35/gcy-36*-expressing neurons.

### GCY-35/GCY-36—TAX-2/TAX-4 signalling pathway mediates ethanol activating O_2_ sensory neurons

**Figure 3:**
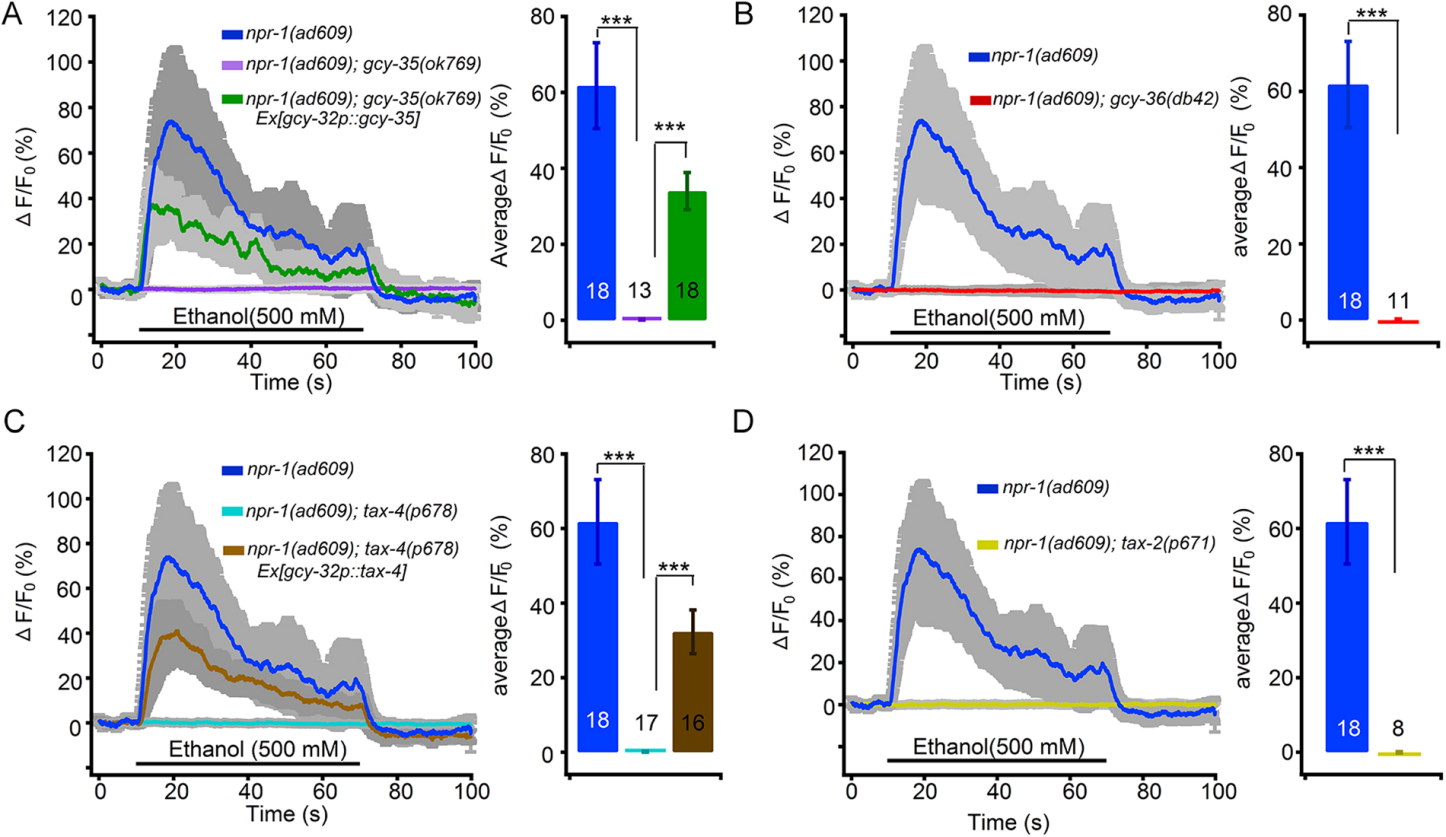
The genes encoding GCY-35/GCY-36 and TAX-2/TAX-4 mediate ethanol-evoked Ca^2+^ responses in O_2_ sensitive neuron URXs. The somal Ca^2+^ transients in URXs in response to ethanol (500 mM) in (**A**) *npr-1*(*ad609*); *gcy-35*(*ok769*), *gcy-32p*::*gcy-35* transgene, etc., (**B**) *npr-1*(*ad609*); *gcy-36*(*db42*), etc., (**C**) *npr-1*(*ad609*); *tax-4*(*p678*), etc., and (**D**) *npr-1*(*ad609*); *tax-2*(*p671*), etc. One-way ANOVA. In (**A**), *F* (2, 46) = 14.17, *P* < 0.0001; in (**C**), *F* (2, 48) = 16.77, *P* < 0.0001. ***p ≤ 0.001 compared as indicated.

To examine the role of the GCY-35/GCY-36—TAX-2/TAX-4 signalling pathway in ethanol activation of O_2_ sensory neurons, we first examined ethanol-evoked URX Ca^2+^ signals in *npr-1*(*ad609*); *gcy-35*(*0k769*) double mutants and the *gcy-35* neuron-specifically reconstituted transgene. URXs exhibited robust Ca^2+^ signals in response to 500 mM ethanol in the *npr-1*(*ad609*) worms and almost no response in the *npr-1*(*ad609*)*; gcy-35*(*ok769*) worms (Fig. 3A). Notably, transgenic rescue of *gcy-35* in three O_2_-sensitive neurons by extrachromosomal expression of the cDNA driven by the *gcy-32* promoter markedly restored the Ca^2+^ signals in URXs (Fig. 3A). We next examined URX Ca^2+^ signals in acute ethanol exposed *npr-1*; *gcy-36* double mutants. As expected, URXs did not show obvious ethanol-evoked Ca^2+^ transients in *npr-1*(*ad609*)*; gcy-36*(*db42*) (Fig. 3B) and *npr-1*(*ad609*)*; gcy-36*(*db66*) (Supplementary Fig. 5A), similar to the response in the *npr-1; gcy-35* double mutant (Fig. 3A). Collectively, these data support that the GCY-35/GCY-36 complex is necessary for neuronal excitation by ethanol in URXs.

The cGMP-gated TAX-2/TAX-4 channel acts downstream of the sGC GCY-35/GCY-36 complex. For testing the role of the channel in URX activation by ethanol, we used *npr-1; tax-2* and *npr-1*; *tax-4* mutants to examine Ca^2+^ responses to ethanol in the neurons. URXs displayed no visible increase in Ca^2+^ transients in *npr-1*(*ad609*); *tax-4*(*p678*) (Fig. 3C), *npr-1*(*ad609*); *tax-2*(*p671*) (Fig. 3D), and *npr-1*(*ad609*)*; tax-4*(*ks28*) (Supplementary Fig. 5B), similar to URX responses in *npr-1*; *gcy-35* and *npr-1*; *gcy-36* double mutants (Fig. 3A,B, Supplementary Fig. 5A). The extrachromosomal expression of *tax-4* cDNA driven by the *gcy-32* promoter obviously restored URX robust Ca^2+^ responses to ethanol (Fig. 3C).

The Ca^2+^ responses of URXs in *npr-1(ad609)* were well related with ethanol concentrations from 50 mM to 1.5 M (Fig. 2E). To determine whether the GCY-35/36—TAX-2/TAX-4 signalling pathway is also indispensable for URX responses to a high concentration of ethanol in *npr-1* animals, we recorded the Ca^2+^ transients in URXs in response to 1.5 M ethanol in the double mutants *npr-1*(*ad609*); *gcy-35*(*0k769*), *npr-1*(*ad609*); *tax-4*(*p678*) and *npr-1*(*ad609*)*; tax-2*(*p671*). Our result showed that all the double mutants displayed no visible ethanol-evoked URX Ca^2+^ signals (Supplementary Fig. 6A–C). The extrachromosomal expression of *gcy-35* and *tax-4* cDNA driven by the *gcy-32* promoter markedly restored URX robust Ca^2+^ responses to ethanol in *npr-1*(*ad609*); *gcy-35*(*0k769*) (Supplementary Fig. 6A) and *npr-1*(*ad609*)*; tax-4*(*p678*) (Supplementary Fig. 6B), respectively. These data demonstrate that the GCY-35/GCY-36—TAX-2/TAX-4 signalling pathway mediates ethanol activation of O_2_ sensory neurons over a wide range of concentrations.

To test whether the GCY-35/GCY-36 complex is an ethanol receptor, we ectopically co-expressed *gcy-35* and *gcy-36* cDNA in the neurons that express *tax-2*/*tax-4* and no *gcy-35*/*gcy-36*. We first tested whether the candidate neurons respond to ethanol. We found that ASIs and ASGs displayed no ethanol-evoked Ca^2+^ signals; however, ASKs and BAGs exhibited ethanol-elicited Ca^2+^ responses (Supplementary Fig. 7). Thus, ASIs and ASGs were chosen for our test. Unexpectedly, ectopic co-expression of *gcy-35* and *gcy-36* did not confer ethanol sensitivity on ASIs or ASGs (Supplementary Fig. 7). Currently, we cannot exclude that this was due to a technical failure. Whether this complex is an ethanol receptor still needs further study.

### Activation of the O_2_-sensory neurons URXs, AQR and PQR is indispensable for acute functional ethanol tolerance

**Figure 4:**
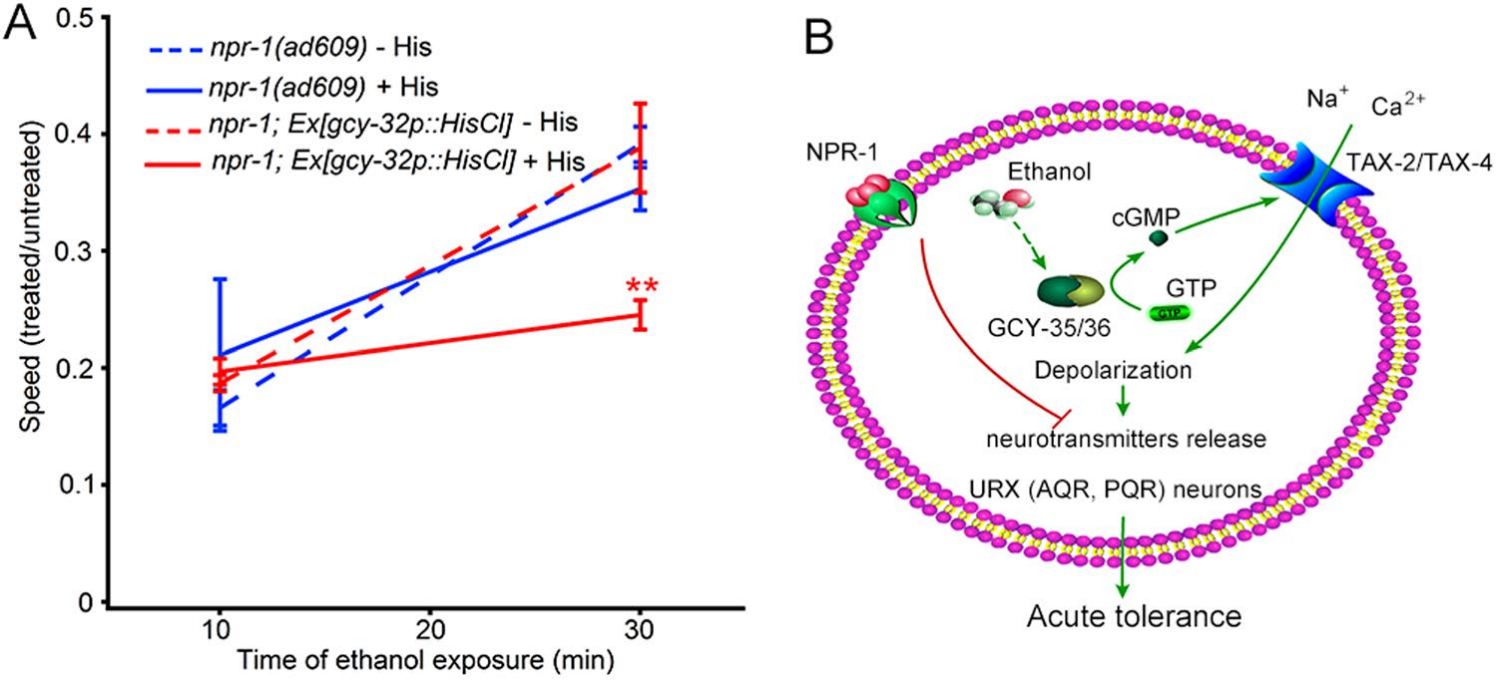
Activation of O_2_ sensory neurons URXs, AQR and PQR is required for acute functional ethanol tolerance in *npr-1* worms. (**A**) Chemogenetic inhibition of URXs, AQR and PQR suppresses the acute functional ethanol tolerance in animal. Two-way ANOVA. *F*_Time_ (1, 62) = 54.02, *P* < 0.0001; *F*_genotype_ (3, 62) = 2.050, *P* = 0.1160; *F*_genotype * Time_ (3, 62) = 4.180, *P* = 0.0093. Significant posttests difference at 30 min in comparisons with mutant with or without histamine treatment, or the transgene without histamine application is the same, and indicated as **p ≤ 0.01. (**B**) Working model for the molecular basis of acute functional ethanol tolerance.

To verify the role of the O_2_ sensory neurons in acute functional ethanol tolerance, we used chemogenetics to silence their activities. We employed specific expression of *Drosophila HisCl1* encoding a histamine-gated chloride channel driven by the *gcy-32* promoter in *npr-1*(*ad609*) (Supplementary Fig. 8) and administration of 10 mM histamine^47^ to chemogenetically inhibit the neurons. Our result showed that chemogenetically silencing O_2_ sensory neurons markedly suppressed *npr-1* acute functional ethanol tolerance without impacting worm sensitivity to ethanol (Fig. 4A), although *HisCl1* expression in O_2_-sensitive neurons alone (without histamine treatment) in N2 worms also suppressed acute functional ethanol tolerance (Supplementary Fig. 9). This is possibly a result of the low probability of opening the HisCl1 channel without the ligand histamine or being activated by unknown ligands in worms. Taken together, these data suggest that activation of URX, AQR and PQR neurons is required for acute functional ethanol tolerance in *npr-1* worms.

In summary, in this study, we presented data supporting that the sGC heterodimer GCY-35/GCY-36—cGMP-gated channel (TAX-2/TAX-4) signalling pathway in O_2_ sensory neurons mediates the acute functional ethanol tolerance in *npr-1* worms and that ethanol directly activates these neurons. The hypothesized molecular basis is shown in Fig. 4B.

## Discussion

*C. elegans* lives in rotting fruit and vegetation that contain lower oxygen levels than that in air. The low oxygen level may protect the worms from the damage of reactive oxygen species^25,48,49^. *C. elegans* develops O_2_ sensation and moves faster under high O_2_ levels^25,30,34^. Due to metabolic pathways of bacteria, bacterial flourishing produces alcohol and reduces ambient oxygen levels in microenvironments. The presence of alcohol and a low oxygen level means a sufficient supply of bacterial food for worms. However, high levels of alcohol make worms intoxicated and uncoordinated. The nematodes develop sensations and behavioural responses to oxygen and ethanol. This endows worms with advantages for survival and population propagation in the wild. Loss-of-function mutations and the lower function 215F natural allele of NPR-1 make worms ethanol resistant (Supplementary Fig. 2D), which is consistent with a previous report^23^. The high level of ethanol (500 mM)-treated *npr-1* animals move as fast at 7% O_2_ as at 21% O_2_ (Supplementary Fig. 2). Possible explanations are as follows: URXs display an increase in Ca^2+^ signals in response to the switch of O_2_ from 7% to 21%, similar to those evoked by 500 mM ethanol, as shown in Supplementary Fig. 10, and the O_2_ sensory neurons respond to these two stimuli almost equally. However, the response is possibly not additive. The aversive responses to the harmful stimuli of high levels of oxygen and acute functional tolerance to high levels of ethanol endue worms to avoid harmful environments, and these two functions possess similar adaptive significance.

The cGMP signalling pathway is involved in sensations and many physiological activities^50^. In *C. elegans*, it plays essential roles in the chemosensation of soluble and volatile compounds^32^, oxygen^25,30^ and CO_2_^51^, as well as in photosensation^52^ and water sensation^31^. It is also involved in long-term physiological processes, including satiety, lifespan, lethargy, innate immunity and development^53–56^. GCY-35 and GCY-36, consisting of a sGC complex that is expressed in sensory neurons URX, AQR and PQR, are required in URX sensory neurons to sense increases in O_2_ levels and affect corresponding behaviours^25,30,34^. The heme-binding domain of GCY-35 binds molecular oxygen, NO and CO^25,57^. Is the GCY-35/GCY-36 complex an ethanol receptor? Unfortunately, calcium responses to various concentrations of ethanol could not be well fitted by the Hill function, and ectopic co-expression of both *gcy-35* and *gcy-36* cDNA in ASI or ASG neurons that expressed *tax-2* and *tax-4* was not able to induce a Ca^2+^ response to ethanol, albeit technical failure cannot be excluded so far. These results hint that the complex is unlikely to be an ethanol receptor. This open question needs further study. However, overall, the present study demonstrates that GCY-35/GCY-36—TAX-2/TAX-4 signalling mediates the ethanol excitatory effect on O_2_ sensory neurons and acute worm function in ethanol tolerance. As many molecular mechanisms in neuronal activities are conserved in the animal kingdom, our finding adds a new and important aspect to the cGMP signalling pathway and provides a new clue to understanding the complicated mechanisms of alcohol effects.

## Methods

### Assays of worm behavioral responses to acute ethanol treatment

The ethanol effect on worm locomotion was tested as described^6,23,27^, with minor modifications. In short, 10 ml of hot 2% nematode growth media (NGM) was poured into a 6 cm diameter Petri dish and dried for 2 hour at 37 °C. Anhydrous ethanol was added onto the test plate unseeded with bacterium to achieve the final concentration of 500 mM used in the behavioral tests. The dish was covered with lid, sealed with Parafilm and stored for more than 2 hour at room temperature to allow ethanol diffuse. 10 young adult worms were transferred into a plate unseeded with bacterium and allow them to move freely for 30 min, then were transferred onto an area that was circled by a filter paper ring (inner diameter 1.5 cm) soaked with 20 mM CuSO_4_ solution to limit worm exploration range on agar plates added with or without ethanol for ethanol treated or untreated locomotion test. Worm locomotion was recorded under a Zeiss Discovery V8 stereomicroscope (Carl Zeiss MicroImaging GmbH, Göttingen, Germany). The image sequences were captured with an Andor iXon^EM^ + DV885K EMCCD camera (Andor Technology plc., Springvale Business Park, Belfast, UK) at 1 frame per second. The worm locomotion speed was analyzed by use of multi-worm Tracker software^58^. The relative speeds were calculated as: Speed ratio = speed _ethanol treated_/speed _ethanol untreated_. The relative locomotion speed during period of 2 min at 10 and 30 min after worm exposure to ethanol were used as indicators for worm sensitivity and functional tolerance to ethanol, respectively.

### Chemogenetics

A chemogenetic method was used to inhibit neuron activity as described^47^. In short, a *Drosophila* chloride channel, HisCl1, was ecto-expressed specifically in the sensory neurons URXs, AQR and PQR driven by *gcy-32* promoter, and 10 mM histamine was applied to activate the channel. For making the histamine-NGM agar plates, we added 1 M histamine dihydrochloride (Sigma-Aldrich, ST Louis, USA) stock solution (in ultra-pure water) into NGM agar at ∼65 °C immediately before pouring plates.

### Data analysis

Data statistical analysis was conducted by use of GraphPad Prism 6 (GraphPad Software, Inc., CA, USA). We used one-way analysis of variance to test the mean values among three or more than three samples with single factor, and used two-way analysis of variance to determine the significant difference between groups for two factors (time and genotypes). Next, we used *Bonferroni*’s *t*-test correction for multiple comparisons. Results are presented as the mean values ± SEMs with number of experimental replications (n).

## Electronic supplementary material

Supplementary Movie 1. N2 treated

Supplementary Movie 2. N2 untreated

Supplementary Movie 3. npr-1 treated

Supplementary Movie 4. npr-1 untreated

Supplementary Movie 5. npr-1 gcy-35 treated

Supplementary Movie 6. npr-1 gcy-35 untreated

Supplementary Movie 7. npr-1 gcy-35 rescue treated

Supplementary Movie 8. npr-1 gcy-35 rescue untreated

Supplementary Information
