# Supplementary material for: GCY-35/GCY-36—TAX-2/TAX-4 Signalling in O2 Sensory Neurons Mediates Acute Functional Ethanol Tolerance in Caenorhabditis elegans
Source: Sci Rep. 2018 Feb 14;8:3020. doi: 10.1038/s41598-018-20477-z (PMC5813177; doi:10.1038/s41598-018-20477-z)
Supplement: Supplementary file 9 — Supplementary Information [file 41598_2018_20477_MOESM9_ESM.pdf]

GCY-35/GCY-36—TAX-2/TAX-4 Signalling in O_2 Sensory Neurons Mediates Acute Functional Ethanol Tolerance in *Caenorhabditis elegans*

Yuan-Hua Chen*, Chang-Li Ge*, Hong Wang, Ming-Hai Ge, Qing-Qin He, Yu Zhang, Wei Tian, Zheng-Xing Wu [§]

Key Laboratory of Molecular Biophysics, Ministry of Education, and Department of Biophysics and Molecular Physiology, College of Life Science and Technology, Huazhong University of Science and Technology, Wuhan, 430074, P.R. China

* These authors contributed equally to this work.

[§] To whom correspondence should be addressed. Email: ibbwuzx@mail.hust.edu.cn

Supplementary Materials and Methods

Strains. All strains were maintained and grown under standard conditions[59]. The wild type worm was *C. elegans* Bristol N2. The double mutants used in this study were generated by use of the standard genetic techniques[59] and confirmed by PCR or sequencing. All transgenic strains were generated using the standard microinjection techniques[60]. Most plasmids were injected at 50 – 70 ng/μl together with *lin-44p::gfp* (10 ng/μl) as a co-injection marker. *C.*

elegans strains used in this study are listed in Supplementary Table 1.

Molecular Biology. The most expression constructs were generated with the Three-Fragment Multisite Gateway® system (Invitrogen™, Thermo Fisher Scientific, Waltham, MA, USA). Briefly, three entry clones comprising three PCR products (promoter, gene of interest, *sl2::GFP* or *3' UTR*, in name of slot1, slot2 and slot3, respectively) were recombined into the pDEST™ R4-R3 Vector II or custom-modified destination vectors using *att*L-*att*R (LR) recombination reactions to generate the expression clones. *gcy-35* cDNA was amplified by RT-PCR from *C. elegans* mixed stage RNA. *tax-4* cDNA was amplified from a plasmid kindly gifted by Dr. Mario de Bono. The following promoters were used to drive the test genes express specifically in the target neurons respectively: a 0.7 kb *gcy-32* promoter in AQR, PQR and URXs[29], a 2.46 kb *gpa-4* promoter in ASIs[61] and a 2 kb *ops-1* promoter in ASGs[62]. The *tax-2p*::*gcy-36* plasmid was constructed by replacing the *flp-17* promoter fragment between *SalI* and *KpnI* sites in *flp-17p*::*gcy-36* expression plasmid[30] (a gift from Dr. C. Bargmann) with a *tax-2* promoter fragment.

Calcium Imaging. The calcium transients in the soma and axon of neurons examined were measured by detecting changes in the fluorescence intensity of R-GECO1, a sensitive and rapid kinetic calcium indicator of weak photobleaching[36]. A homemade PDMS microfluidic device was used to trap worms and to deliver solutions[37,38,63]. In detail, a young adult worm was

transferred from OP50 lawn into a drop of M13 buffer solution to wash bacteria off its body. Then, the animal was loaded into the worm channel in the microfluidic chip with its nose exposed to buffer, solutions or gas under laminar flow. Ethanol solutions in M13 buffer and others were delivered via a programmable automatic drug-feeding equipment (MPS-2, InBio Life Science Instrument Co. LTD, Wuhan, China). R-GECO1 was excited by 525 nm – 530 nm light emitted by an Osram Diamond Dragon LTW5AP light-emitting diode (LED) model (Osram, Marcel-Breuer-Straße 6, Munich, Germany) constructed in a multi-LED light source (MLS102, InBio Life Science Instrument Co. LTD) and filtered with a Semrock FF01-593/40-25 emission filter (Semrock, Inc., NY, USA), under an Olympus IX-70 inverted microscope (Olympus, Tokyo, Japan) equipped with a 40 × objective lens (numerical aperture (NA) = 1.3, Zeiss). Fluorescence images were captured with an Andor iXon^{EM}+ DU885K EMCCD camera with a 100 ms exposure time and 256 × 256 pixels at 10 frames per second. Each animal was recorded once only. The averaged fluorescence intensity of the region of interest (ROI) of the soma was captured and analyzed by use of Image-Pro Plus 6.0 (Media Cybernetics Inc, Rockville, MD, USA). An adjacent ROI in each frame was used to subtract background. The average fluorescence intensity within the initial 10 s before stimulation was taken as basal signal F_0. The percent changes of fluorescence intensities relative to the initial intensity F_0, $\Delta F / F_0 = (F - F_0) / F_0 \times 100$ %, were plotted as a function of time for all curves. The mean values of Ca^{2+} signals and the SEMs were

plotted in various colors as indicated and in light gray respectively by use of IGOR Pro 6.10 (Wavemetrics, Portland, OR, USA).

Making a Low [O_2] Test Micro-environment

We used a 9 cm (in diameter) Petri dish to make a low oxygen aerial environment and a 6 cm dish inside it as test plate to examine the effect of different oxygen levels on worm locomotion. In detail, a 9 cm dish was drilled two holes on the opposite and insert two tubes for gas inlet and outlet respectively, then covered with lid and sealed with Parafilm. The assay dish (6 cm in diameter) was covered with lid and sealed with Parafilm that was cut an 1 cm gap along the edge of lid for interchanging gas with the outer dish, blocking the gas flow to affect worms and retarding volatilization of ethanol (Supplementary Fig. 2A). A 7 % oxygen plus 93 % nitrogen mixed gas was infused into the 9 cm dish for more than 10 min before and during the whole locomotion recording in the behavioral tests. We examined the oxygen levels in the 6 cm dish using 0.5 mM (dissolved in ethanol) O_2 sensitive fluorescent dye $Ru(phen)_3Cl_2$ (Sigma-Aldrich). The fluorescence of the compound increases with the decrease of oxygen levels[30,35]. As shown in (Supplementary Fig. 2B), the fluorescence reach to the maximum at about 12 minutes in our test condition, indication that the oxygen level decrease to about 7 % in 12 minutes via infusion of the 7 % oxygen gas. On plates seeded with the bacteria OP50, the *npr-1*(*ad609*) mutant moved significantly slower in low [O_2] than in air (Supplementary Fig. 2C), in agreement with previous report[23], verifying that

this simple device worked well for the tests in the low oxygen levels.

Measurement of Dissolved Oxygen in Ethanol Solutions

We added various amounts of dehydrated ethanol into M13 buffer to get solutions with different levels of ethanol. We used an electronic dissolved oxygen meter (Sinomeasure, Hangzhou, China) to measure the saturated dissolved oxygen levels in the solutions. The measurement is oxygen-consumed process. Therefore, we vibrated the buffer to maintain oxygen saturation during the measurement process.

References

59 Brenner, S. The genetics of *Caenorhabditis elegans*. *Genetics* **77**, 71-94 (1974).

60 Mello, C. C., Kramer, J. M., Stinchcomb, D. & Ambros, V. Efficient gene transfer in *C. elegans*: extrachromosomal maintenance and integration of transforming sequences. *EMBO J.* **10**, 3959-3970 (1991).

61 Jansen, G. *et al.* The complete family of genes encoding G proteins of *Caenorhabditis elegans*. *Nat. Genet.* **21**, 414-419, doi:10.1038/7753 (1999).

62 Juozaityte, V. *et al.* The ETS-5 transcription factor regulates activity states in *Caenorhabditis elegans* by controlling satiety. *Proc. Natl. Acad. Sci. USA* **114**, 1651-1658, doi:10.1073/pnas.1610673114 (2017).

63 Wang, W. *et al.* Off-response in ASH neurons evoked by $CuSO_4$ requires the TRP channel OSM-9 in *Caenorhabditis elegans*. *Biochem. Biophys. Res. Commun.* **461**, 463-468, doi:10.1016/j.bbrc.2015.04.017 (2015).

Supplementary Table

Supplementary Table 1 | Mutant and transgenic worms used in the study.

Strains	Genotypes
N2	Bristol wild-type
CB4856	wild-type isolated from Hawaii
DA609	*npr-1*(*ad609*) X
CX7157	*npr-1*(*ad609*) X; *gcy-35*(*ok769*) I

CX4819	*npr-1*(*ad609*) X; *tax-4*(*p678*) III
AX1295	*gcy-35*(*ok769*) I
CX6448	*gcy-35*(*ok769*) I
AX1296	*gcy-36*(*db42*) X
FK104	*tax-2*(*ks31*) *I*
PR694	*tax-2*(*p694*) *I*
PR678	*tax-4*(*p678*) *III*
ZXW1001	*npr-1*(*ad609*) X; *hkdEx1001*[*gcy-32p::RGECO1; lin-44p::GFP*]
ZXW1002	*hkdEx1002*[*gcy-32p::RGECO1; lin-44p::GFP*]
ZXW1003	*npr-1*(*ad609*) X; *hkdEx1003*[*gcy-32p::HisCl1::sl2::GFP; lin-44p::GFP*]
ZXW1004	*gcy-35*(*ok769*) I; *npr-1*(*ad609*) X; *hkdEx1004*[*gcy-32p::gcy-35::sl2::GFP; lin-44p::GFP*]
ZXW1005	*gcy-35*(*ok769*) I; *npr-1*(*ad609*) X; *hkdEx1005*[*gcy-32p::RGECO1; lin-44p::GFP*]
ZXW1006	*gcy-35*(*ok769*) I; *npr-1*(*ad609*) X; *hkdEx1006*[*gcy-32p::gcy-35::sl2::GFP; gcy-32p::RGECO1; lin-44p::GFP*]
ZXW1008	*npr-1(ad609)* X; *tax-4*(*p678*) III; *hkdEx1008*[*gcy-32p::tax-4::sl2::GFP; gcy-32p::RGECO1; lin-44p::GFP*]
ZXW1012	*gcy-36(db66) X; npr-1(ad609) X; hkdEx1012 [gcy-32::RGECO1; lin-44p::GFP]*
ZXW1014	*tax-2*(*p671*) I; *npr-1*(*ad609*) X
ZXW1015	*tax-2*(*p671*) I; *npr-1*(*ad609*) X; *hkdEx1015*[*gcy-32p::RGECO1; lin-44p::GFP*]
ZXW1018	*npr-1*(*ad609*) X; *gcy-36*(*db42*) X
ZXW1019	*npr-1*(*ad609*) X; *gcy-36*(*db42*) X*; hkdEx1019*[*gcy-32p::RGECO1; lin-44p::GFP*]
ZXW1030	*unc-13*(*e1091*) I; *hkdEx1030*[*gcy-32p::RGECO1; lin-44p::GFP*]
ZXW1031	*unc-31*(*e928*) IV; *hkdEx1031*[*gcy-32p::RGECO1; lin-44p::GFP*]
ZXW1032	*hkdEx1032*[*gpa-4p::gcy-35::sl2::GFP; tax-2p::gcy-36; gpa-4::RGECO1*]
ZXW1033	*hkdEx1033*[*ops-1p::gcy-35::sl2::GFP*; *tax-2p::gcy-36*; *ops-1p::RGECO1*]
ZXW1034	*hkdEx1034*[*gcy-32p::hiscl1::sl2::GFP*]
ZXW1035	*npr-1*(*ad609*) X; *tax-4*(*ks28*) III; *hkdEx1035*[*gcy-32p::RGECO1*; *lin-44p::GFP*]
ZXW1036	*hkdEx1036*[*sra-9p::RGECO1; lin-44p::GFP*]
ZXW1037	*hkdEx1037*[*flp-17p::RGECO1; lin-44p::GFP*]

Supplement Figure

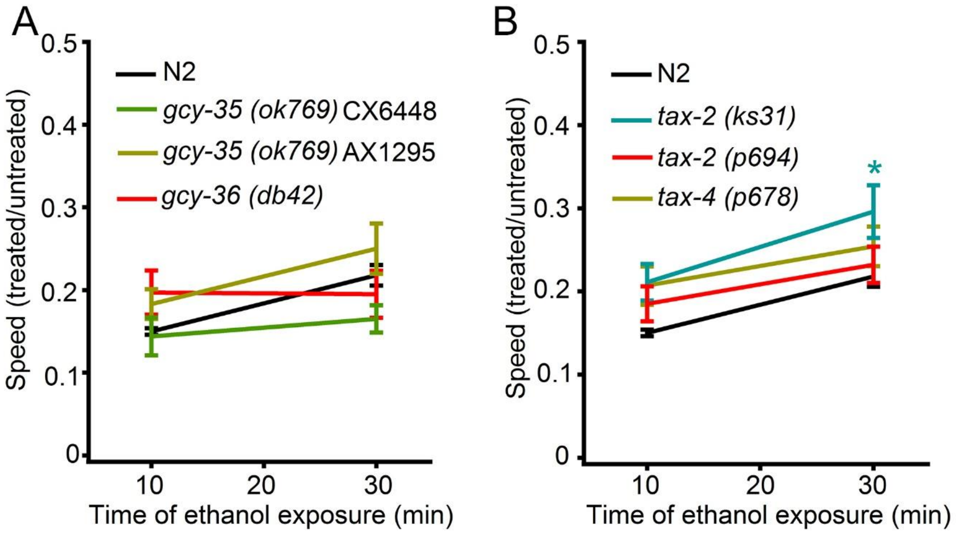

Supplementary Figure 1. *gcy-35/36 and tax-2/tax-4* mutants displayed no significant difference in sensitivity and acute functional tolerance to ethanol.

A and **B** The relative locomotion speed (ethanol treated/untreated) at 10 and 30 min after exposure to ethanol (500 mM) in (A) wild type N2*, gcy-35(ok769)*, *gcy-36(db42)* mutants, (B) *tax-2(ky31), tax-2(p694), and tax-4(p678)* mutants. The data are analyzed by two-way ANOVA. In A**,** F_{Time} (1, 34) = 3.984, P = 0.0540; F_{genotype} (3, 34) = 1.406, P = 0.2579; $F_{\text{genotype * Time}}$ (3, 34) = 1.002, P = 0.4040; in B, F_{Time} (1, 36) = 12.79, P = 0.0010; F_{genotype} (3, 36) = 3.227, P = 0.0337; $F_{\text{genotype * Time}}$ (3, 36) = 0.2743, P = 0.8435. Significant posttests differences compared with N2 at 30 min time point are indicated (* $p \le 0.05$).

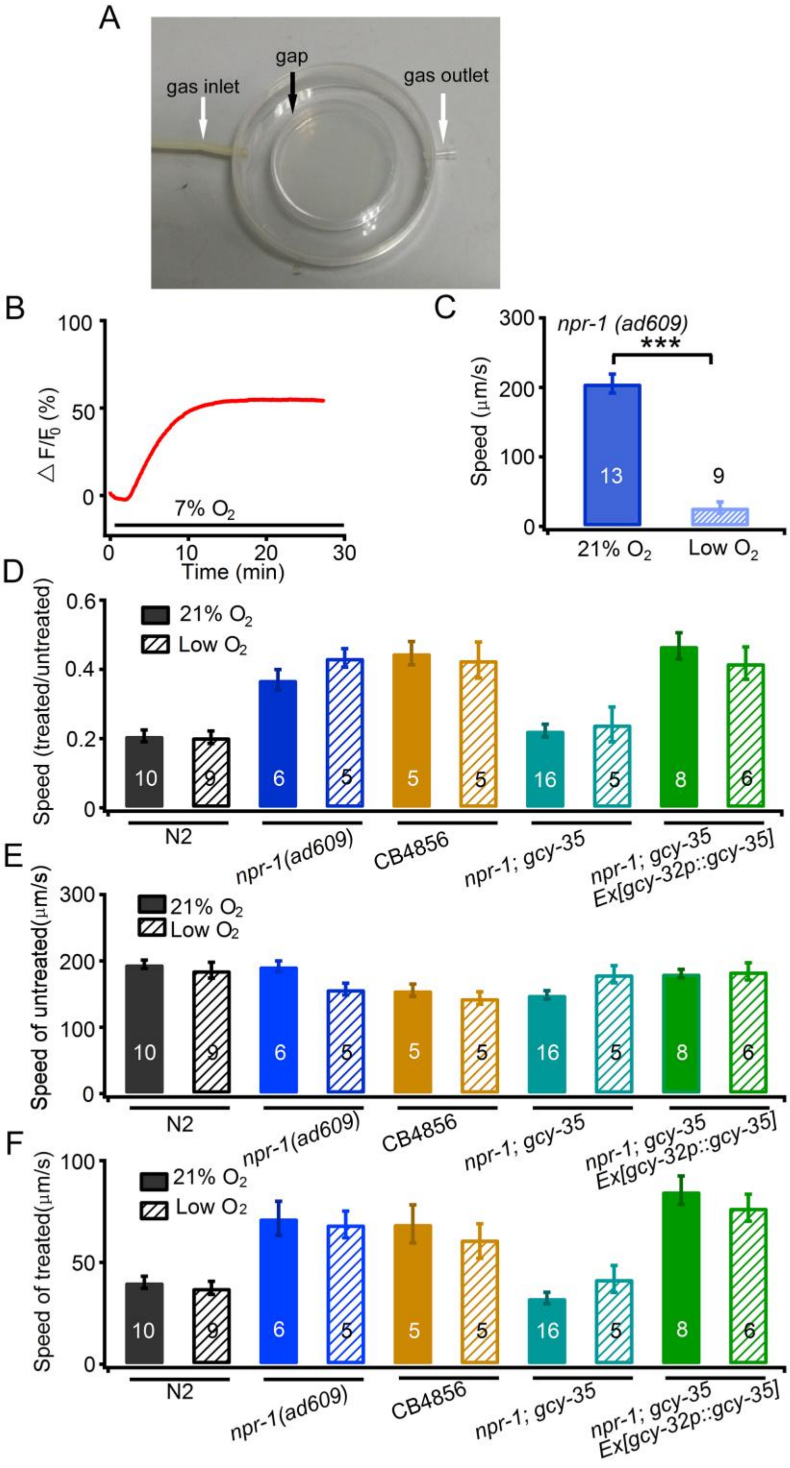

Supplementary Figure 2. Acute functional ethanol tolerance in worms is independent of oxygen levels.

A The device used for behavioural tests under low [O_2] condition, which is described in detail in methods.

B Fluorescence changes of the O_2-sensitive dye Ru(phen)$_3$Cl$_2$ dissolved in assay plate during infusion of 7 % oxygen + 93 % nitrogen mixed gas.

C A low O_2 level decreased *npr-1(ad609)* worm locomotion speed on the plate seeded with OP50 bacteria. *** $p \le 0.001$ compared with that of *npr-1*(*ad609*) worms in 21 % O_2 level.

D Relative Locomotion speeds (treated/untreated) in N2 (215V), *npr-1*(*ad609*), CB4856 (215F), *npr-1*(*ad609*); *gcy-35(ok769)* and the transgenes indicated in different ambient O_2 levels, respectively.

E Locomotion speeds in food deprived worms without ethanol treatment in different ambient O_2 levels.

F Locomotion speeds of food deprived worms after ethanol exposure (500 mM for 30 min) in different ambient O_2 levels respectively.

All data are expressed as means ± SEMs. The number on each bar indicates the number of independent tests for each genotype.

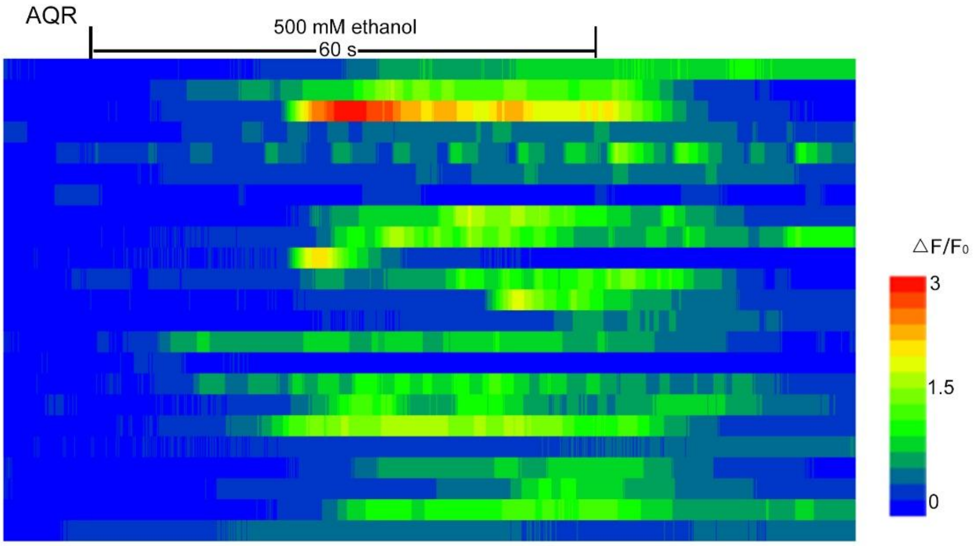

Supplementary Figure 3. Heatmap of calcium dynamics in AQR neuron in *npr-1(ad609)* mutant during exposure to 1-min pulse stimulation of 500 mM ethanol.

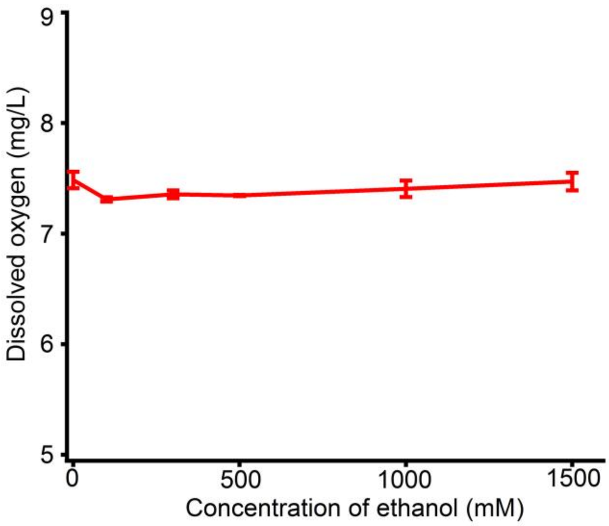

Supplementary Figure 4. Dissolved oxygen levels in ethanol-M13 solutions with different ethanol concentrations.

There are no significant changes of dissolved O_2 levels in various solution.

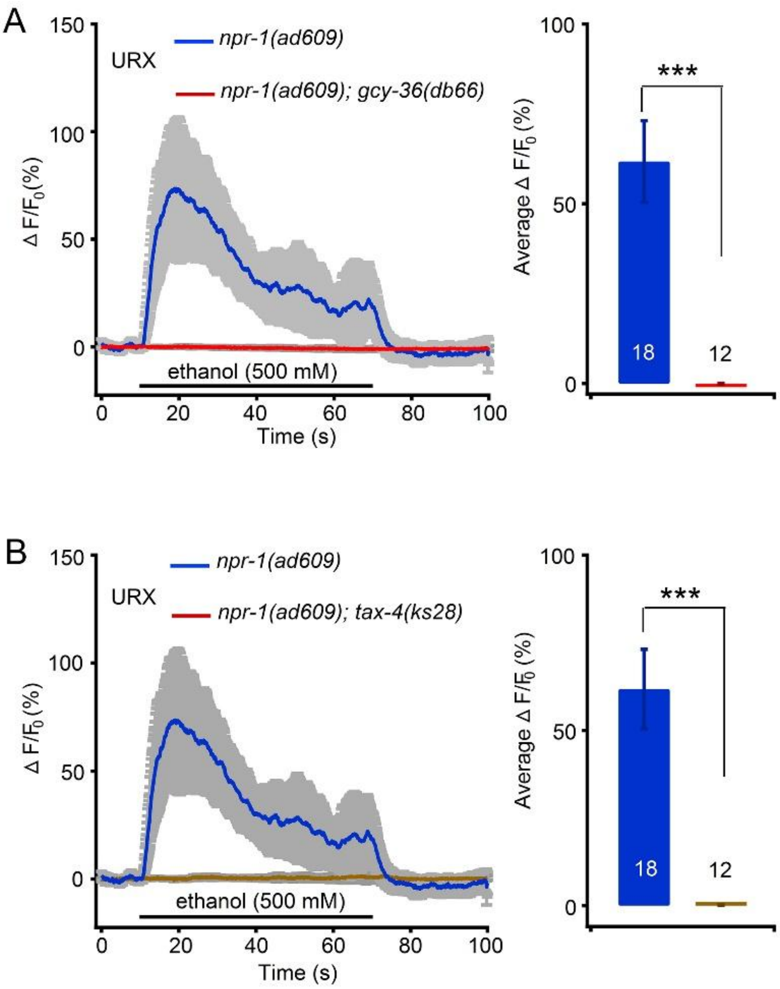

Supplementary Figure 5. *gcy-36* and *tax-4* loss-of-function mutations destroyed the Ca^{2+} response to 500 mM ethanol in *npr-1* worms.

Ethanol (500 mM) elicited URX somal Ca^{2+} responses in the *npr-1*(*ad609*), *npr-1*(*ad609*)*; gcy-36*(*db66*) (A) and *npr-1*(*ad609*)*; tax-4(ks28)*, etc. (B). *** $p \leq 0.001$ compared as indicated.

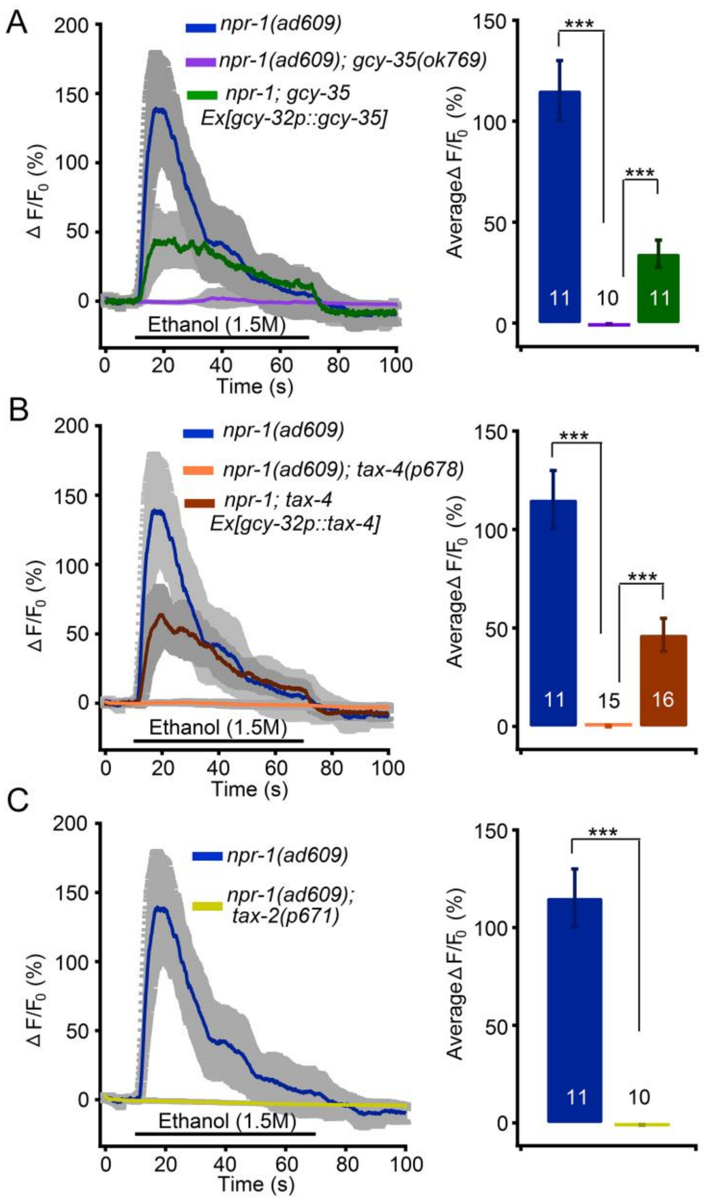

Supplementary Figure 6. The genes encoding GCY-35 and TAX-2/TAX-4 are essential for URXs Ca^{2+} responses to high concentration ethanol in worms.

Ethanol (1.5M) elicited URX somal Ca^{2+} responses in (A) *npr-1(ad609); gcy-35(ok769)*, g*cy-32p::gcy-35* transgene, etc., (B) *npr-1*(*ad609*)*; tax-4(ks28)* **,** etc., and (C) *npr-1*(*ad609*); *tax-2*(*p671*), etc..

One-way ANOVA. In A, F (2, 29) = 37.52, p < 0.0001; in B, F (2, 39) = 40.10, p < 0.0001. *** $p \le 0.001$ compared as indicated.

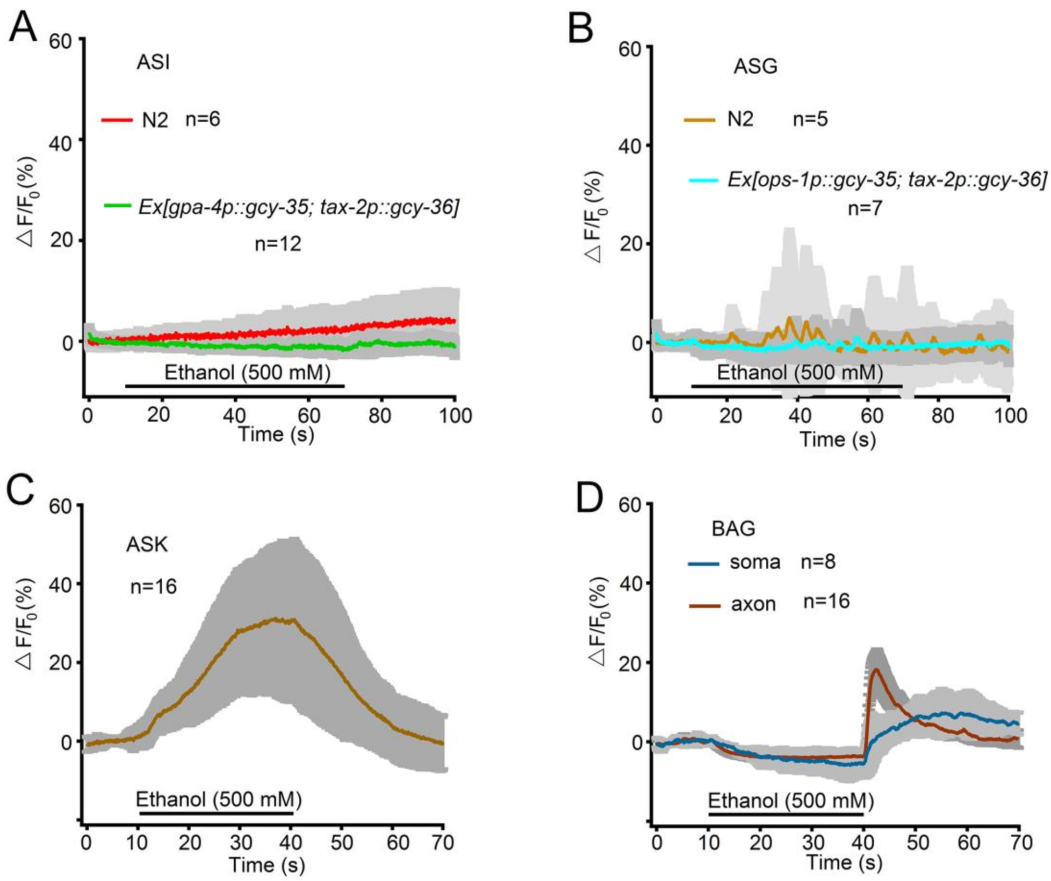

Supplementary Figure 7. The Somal or axonal Ca^{2+} responses to ethanol of neurons indicated in N2 worm and the transgenes.

Ethanol (500 mM) elicited somal Ca^{2+} transients of ASIs (A), ASGs (B) and ASKs(C), somal and axonal Ca^{2+} transients of BAGs in N2 worms (D). The specific extrachromosomal co-expression of *gcy-35* and *R-GECO1.0* cDNA in ASI and ASG neurons which expressing *tax-2* and *tax-4* was directed by the *gpa-4* and *ops-1* promoter, respectively. *gcy-36* cDNA was expressed in ASI, ASG and other neurons driven by a *tax-2* promoter. The specific extrachromosomal expression of *R-GECO1.0* cDNA in ASK and BAG was directed by the *sra-9* and *flp-17* promoter, respectively. The solid traces show the average relative changes of R-GECO1.0 fluorescence ($\Delta F/F_0$) and grey

shadows represent SEMs. The number of independent tests for each genotype is indicated.

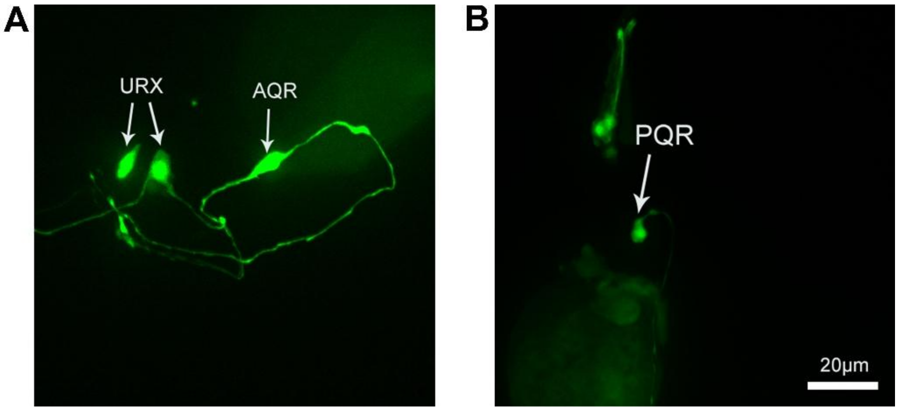

Supplementary Figure 8. Expression of *HisCl1* in URX, AQR and PQR sensory neurons.

Specific extrachromosomal expression of *HisCl1* cDNA in URX, AQR and PQR neurons was directed by the *gcy-32* promoter.

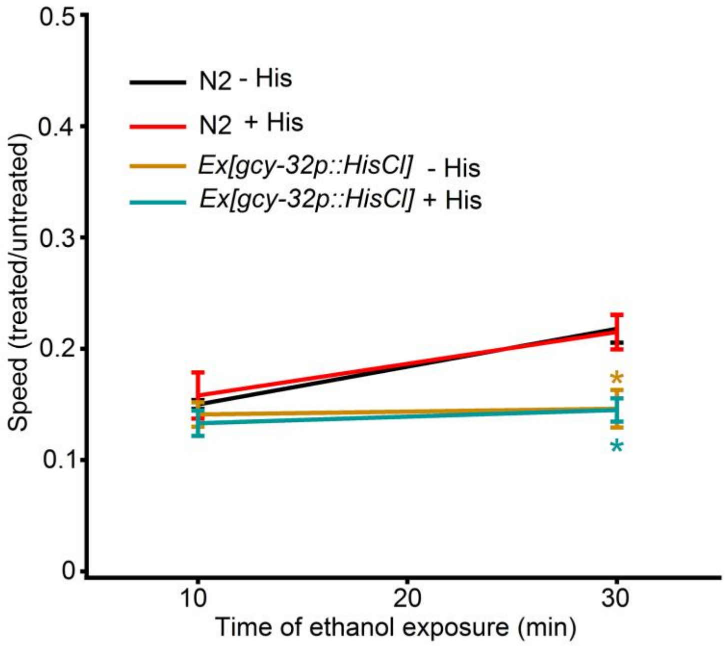

Supplementary Figure 9. Relative locomotion speeds after treatment of ethanol in N2 and the transgene with or without application of exogenous histamine.

Relative locomotion speeds during 2 min at 10 min and 30 min after treatment of ethanol (500 mM), in N2 worm and the transgene with or without application of exogenous histamine (10 mM). Two-way ANOVA, F_{Time} (1, 46) = 6.239, P = 0.0161; F_{genotype} (3, 46) = 3.272, P = 0.0294; $F_{\text{genotype * Time}}$ (3, 46) = 1.245, P = 0.3042, significant posttests differences compared with N2 without Histamine treatment at 30 min time point are indicated (* $p \leq 0.05$).

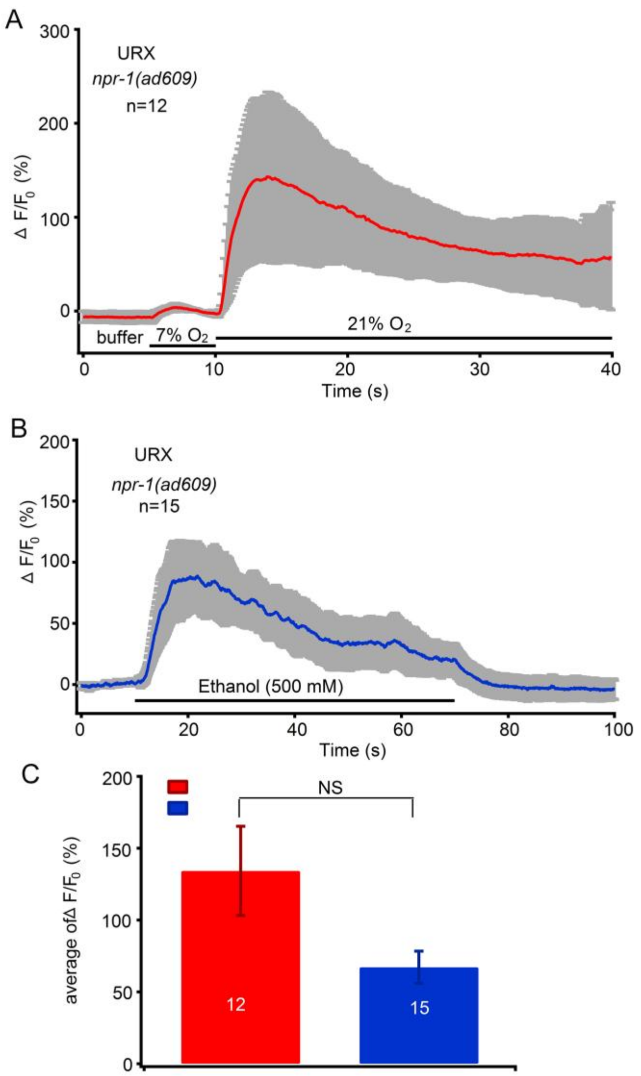

Supplementary Figure 10. URXs displayed similar Ca^{2+} responses to ethanol of 500 mM and the switch of O_2 from 7% to 21%.

A The Ca^{2+} transients in URXs in responses to 7% and 21% O_2, and the switch between O_2 of two levels.

B The Ca^{2+} transients in URXs in responses to stimulation of 500 mM ethanol.

C Summary of increments of peak Ca^{2+} transient (12 – 22 s) in URXs in responses to the switch from 7% O_2 to 21% O_2, and intensity of peak Ca^{2+} signals (12 s – 22 s) in response to 500 mM ethanol. NS, not significant.

Supplementary Video Legends

Supplementary Movie 1. Locomotion of N2 worms treated with 500 mM ethanol on NGM plate unseeded with bacterium. The apparent locomotion in the movie speeds up 10 times.

Supplementary Movie 2. Locomotion of N2 worms without ethanol treatment on NGM plate unseeded with bacterium. The apparent locomotion in the movie speeds up 10 times.

Supplementary Movie 3. Locomotion of *npr-1*(*ad609*) treated with 500 mM ethanol on NGM plate unseeded with bacterium. The apparent locomotion speeds up 10 times.

Supplementary Movie 4. Locomotion of *npr-1*(*ad609*) without ethanol treatment on NGM plate without bacterial food. The apparent locomotion in the movie speeds up 10 times.

Supplementary Movie 5. Locomotion of *gcy-35*(*ok769*); *npr-1*(*ad609*) treated with 500 mM ethanol on NGM plate without bacterial food. The apparent locomotion in the movie speeds up 10 times.

Supplementary Movie 6. Locomotion of *gcy-35*(*ok769*); *npr-1*(*ad609*) without ethanol treatment on NGM plate unseeded with bacterium. The apparent locomotion in the movie speeds up 10 times.

Supplementary Movie 7. Locomotion of *gcy-35*(*ok769*)*;* *npr-1*(*ad609*)*;*

hkdEx1004 [*gcy-32p::gcy-35::sl2::GFP; lin-44p::GFP*] treated with 500 mM ethanol on NGM plate without bacterial food. The apparent locomotion in the movie speeds up 10 times.

Supplementary Movie 8. Locomotion of *gcy-35*(*ok769*)*; npr-1*(*ad609*)*; hkdEx1004* [*gcy-32p::gcy-35::sl2::GFP; lin-44p::GFP*] without ethanol treatment on NGM plate unseeded with bacterium. The apparent locomotion in the movie speeds up 10 times.
